# Modeling the shape and composition of the human body using dual energy X-ray absorptiometry images

**DOI:** 10.1371/journal.pone.0175857

**Published:** 2017-04-19

**Authors:** John A. Shepherd, Bennett K. Ng, Bo Fan, Ann V. Schwartz, Peggy Cawthon, Steven R. Cummings, Stephen Kritchevsky, Michael Nevitt, Adam Santanasto, Timothy F. Cootes

**Affiliations:** 1 Department of Radiology & Biomedical Imaging, University of California San Francisco, San Francisco, California, United States of America; 2 Graduate Program in Bioengineering, University of California San Francisco, San Francisco, California, United States of America; 3 Graduate Program in Bioengineering, University of California, Berkeley, California, United States of America; 4 Department of Epidemiology and Biostatistics, University of California San Francisco, San Francisco, California, United States of America; 5 San Francisco Coordinating Center, California Pacific Medical Center Research Institute, San Francisco, California, United States of America; 6 Department of Epidemiology, University of Pittsburgh, Pittsburgh, Pennsylvania, United States of America; 7 Centre for Imaging Sciences, University of Manchester, Manchester, United Kingdom; Leeds Beckett University, UNITED KINGDOM

## Abstract

There is growing evidence that body shape and regional body composition are strong indicators of metabolic health. The purpose of this study was to develop statistical models that accurately describe holistic body shape, thickness, and leanness. We hypothesized that there are unique body shape features that are predictive of mortality beyond standard clinical measures. We developed algorithms to process whole-body dual-energy X-ray absorptiometry (DXA) scans into body thickness and leanness images. We performed statistical appearance modeling (SAM) and principal component analysis (PCA) to efficiently encode the variance of body shape, leanness, and thickness across sample of 400 older Americans from the Health ABC study. The sample included 200 cases and 200 controls based on 6-year mortality status, matched on sex, race and BMI. The final model contained 52 points outlining the torso, upper arms, thighs, and bony landmarks. Correlation analyses were performed on the PCA parameters to identify body shape features that vary across groups and with metabolic risk. Stepwise logistic regression was performed to identify sex and race, and predict mortality risk as a function of body shape parameters. These parameters are novel body composition features that uniquely identify body phenotypes of different groups and predict mortality risk. Three parameters from a SAM of body leanness and thickness accurately identified sex (training AUC = 0.99) and six accurately identified race (training AUC = 0.91) in the sample dataset. Three parameters from a SAM of only body thickness predicted mortality (training AUC = 0.66, validation AUC = 0.62). Further study is warranted to identify specific shape/composition features that predict other health outcomes.

## Introduction

Global prevalence of diabetes has more than doubled over the past 30 years, affecting nearly 1 in 10 adults, and increasing numbers of children [1, 2]. The largest contributor is type 2 diabetes, linked to dyslipidemia, hypertension, and insulin resistance, collectively referred to as “metabolic syndrome.” Metabolic syndrome accounts for approximately 6–7% of all-cause mortality, 12–17% of cardiovascular disease, and 30–52% of diabetes [3]. Higher Body Mass Index (BMI), a measure of excess weight, was associated with mortality in early studies [4, 5] but is now controversial [6, 7] because more recent work has shown that higher BMI at older age is protective against mortality. However, measures of body shape and central adiposity have been shown to be associated with increased mortality risk. Waist circumference (WC) and its ratio to the hips are more closely related to adverse outcomes than BMI [8–13]. The ratio of trunk-to-leg volume is a strong indicator of diabetes (fifth-to-first quintile odds ratio 6.8) and mortality risk (odds ratio 1.8), independent of BMI and WC [14], showing that more advanced descriptors of body shape accurately indicate metabolic risk beyond traditional measures. We hypothesize that statistical models of the shape and thickness of the whole body will better determine metabolic status and thus mortality risk than existing body shape measures.

Statistical appearance modeling (SAM) [15] has several successful applications including manufacturing [16], handwriting recognition [17], facial recognition [18], and medical imaging of the brain [19], heart [20], eye, liver, lung, kidney, prostate, knees [21], and proximal femur [22, 23]. To date, this powerful technique has not been applied to quantitative DXA body composition scans. We have developed SAM algorithms to analyze pixel-based shape and composition from whole body dual-energy X-ray absorptiometry (DXA) scans [24, 25]. Statistical appearance models from reanalyzed DXA images provide dominant modes of variance of body shape and thickness across a population. The statistical appearance models can be used to investigate associations of body shape and tissue density distribution and demographic (i.e. sex, race, etc.) and clinically-relevant disease outcomes (diabetes, sarcopenia, mortality) to identify those at high disease risk.

In this study, we present the methods to prepare DXA data for analysis, the challenges associated with image registration, and application of the resulting statistical appearance models to estimate mortality risk as a function of body shape.

## Methods

Here we detail the DXA acquisition and image processing algorithms, as well as the statistical appearance modeling techniques. We then describe the statistical analysis of the models to identify and visualize SAM modes strongly associated with clinical variables such as sex and race, as well as mortality status in a sample of older adults.

### DXA scan analysis

In commercial DXA systems, the X-ray attenuation values are used to directly solve for the mass of fat and lean soft tissue. We previously derived relationships from calibration phantom X-ray attenuations to quantify tissue volume and mass at each pixel in whole-body DXA scans [26]. Using custom software developed by the authors in MATLAB (MathWorks, Inc., Natick, MA), we processed the raw low- and high-energy (HE) X-ray attenuation values from a Hologic QDR 4500A densitometer (Hologic, Inc., Bedford, MA) to produce three types of images for this study: (1) total thickness images, capturing the sum thicknesses all tissues in the body; (2) leanness images, defined as the ratio of fat-free (i.e. lean + bone) tissue thickness to total tissue thickness; and (3) R-value images, defined as the ratio of low-energy attenuation to high-energy attenuation. R-value decreases as thickness increases [27] and is used to calculate soft tissue composition (i.e. percent fat). Note that we define thickness here as tissue thickness projected onto the image plane (tissue thickness = tissue mass / tissue density * pixel area) Total thickness is thus generally the sum of the tissue thickness excluding air cavities. It is equivalent to linear path length an X-ray takes through the body.

Raw X-ray attenuation images from the DXA scanner had a resolution of 327 x 150 pixels, at 16-bit pixel depth. Each pixel had spatial dimensions of 2mm x 13mm. All images were upscaled by a factor of 6.5 in the y-direction to have a resulting resolution of 327 x 975 square (2mm x 2mm) pixels. Output thickness and R-value images were exported with 8-bit depth to be compatible with some of the annotation software.

### Image annotation

We defined 82 points on the skin edges as well as bony and soft tissue landmarks. A subset of available images were used to build an semi-automated annotation algorithm based on Constrained Local Model (CLM) methods [28, 29]. The annotator was blinded to participant data. This CLM was then run on each of the remaining R-file training images. Point placements by the algorithm were manually reviewed and corrected by the human annotator where necessary. Differences in patient positioning led to variations in the extremities, which are of limited importance when examining body composition. Thus we created a 52-point extended torso model, which includes the torso, the upper arms and upper legs, but not the forelimbs.

### Statistical appearance modeling

**Fig 1:**
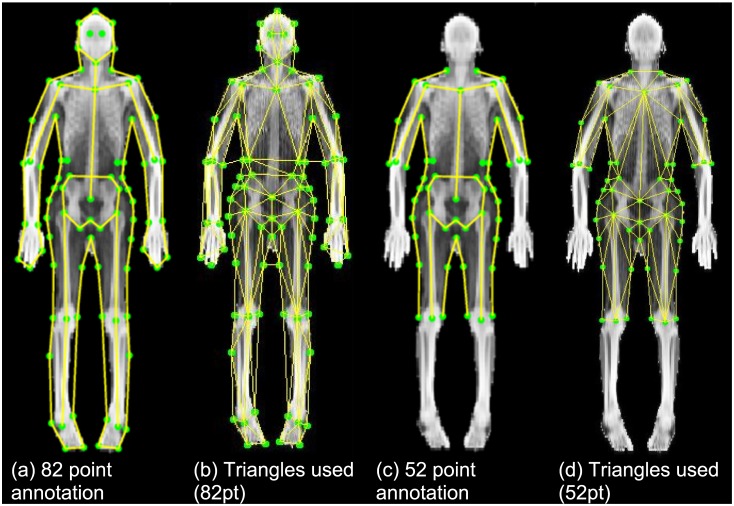
Annotation schemes and triangulations for texture warping. A full-body shape model containing 82 annotation points was initially developed. To eliminate spatial noise introduced by pose variation, a 52-point subset model was created that excludes the forelimbs and head.

**Fig 2:**
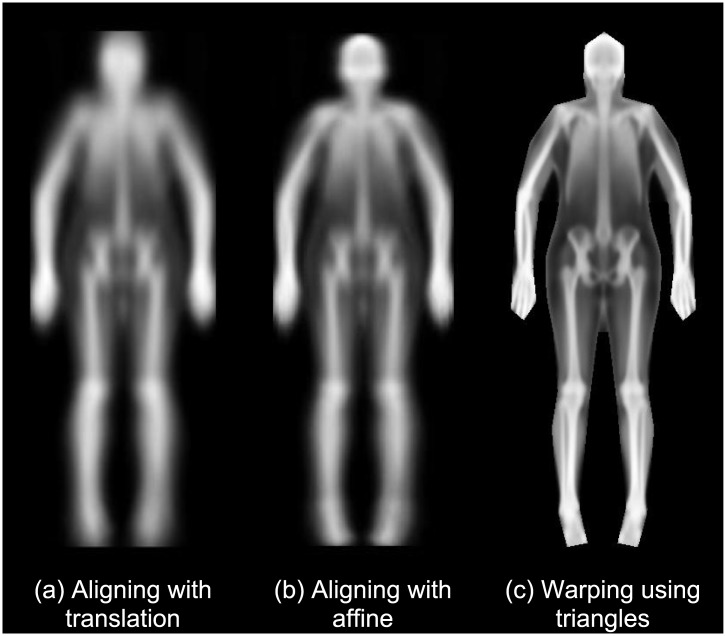
Mean images created with the 200 R-value images. Images were calculated using alignment with (a) translation, (b) translation, rotation, and scaling (affine), and (c) piecewise warping using the triangle model in Fig 1. Each successive mean image is visually sharper than the last, indicating that the more advanced alignment techniques are more effective at eliminating noise from shape and pose variation.

Statistical shape and appearance models were constructed from the annotated images. Details of the approach can be found in [15]. In summary: (1) A shape model is built by (i) translating each set of annotation points so that they have a common center of gravity, (ii) applying Principal Component Analysis (PCA) to vectors containing the 2D annotation point coordinates that represent the aligned shapes for each image. (2) Shape variation is removed by warping each image to a reference frame defined by the mean body shape. Specifically, each image is deformed using a piece-wise affine transformation defined by a triangle mesh (see Figs 1 and 2). (3) A “texture” model is built by applying PCA to vectors defined by the pixel-by-pixel grayscale intensity of these warped images. Texture models contain no 2D (in-plane) shape variation—only grayscale intensity differences due to varying X-ray attenuation measurements for each participant. (4) An “appearance” model is built by applying PCA to vectors formed by concatenating the shape and texture parameters. Appearance models thus capture both shape and texture information and reveal the ways in which shape and texture are correlated.

Concretely, a completed appearance model represents both (in-plane) shape and texture using the linked linear models

$$x=\;\bar{x}+\;Q_{x}c$$

$$g=\;\bar{g}+\;Q_{g}c$$

where *x* is a vector containing the annotation point coordinates, $\bar{x}$ is the mean shape vector, *g* is a vector containing the grayscale pixel intensities in the mean shape reference frame, $\bar{g}$ is the mean grayscale intensity vector, the columns of *Q*_*x*_ and *Q*_*g*_ are the ordered eigenvectors that span the variance in shape and texture across the images, and *c* is the vector of appearance model parameters. We refer to each eigenvector as a mode of shape and texture variation. These modes linearly map the compact parameter vector *c* to the shape and texture vectors *x* and *g*.

The appearance model allows new images with different shapes and textures to be generated by selecting new values for the parameters in *c*. Each image can then be compactly encoded by a vector of parameters,*c*, obtained by fitting a parameter vector *c* that synthesizes an image as close as possible to the original [15].

### Proof of concept sample

A total sample of 400 older adults (ages 70–79) was selected from the longitudinal Health, Aging and Body Composition (Health ABC) study [30–32]. Two sets of 100 cases (participants who died during the first six years of follow-up) and 100 BMI-, sex-, and age-matched controls were selected. One set was used for model calibration and the other was used for validation. Selection was stratified by sex and race (black and white). The Health ABC study was initiated in 1997 by the National Institute on Aging to examine the impact of changes in body composition and health conditions on age-related physiologic and functional status. At baseline, each participant received numerous clinical evaluations including whole body DXA scans acquired using Hologic QDR 4500A systems (Hologic, Inc., Bedford, MA) and software version 9.03, located at two study sites. Validity of fan-beam dual-energy X-ray absorptiometry for measuring fat-free mass and leg muscle mass has been previously reported [33].

Statistical appearance models were trained on the calibration dataset and validated on the validation dataset. We investigated the bivariate association of the SAM parameter vectors to continuous variables of BMI and age using general linear regression models (proc GLM), and categorical variables of mortality status, sex, and race using logistic regression (proc LOGISTIC). Stepwise selection for the most significant SAM parameters, i.e. the number the explained 95% of the variance, were used to select parameters at a significance of *p* ≤ 0.05 to estimate each outcome variable. All statistical analysis was done using SAS software, version 9.2 (SAS Institute, Inc., Cary, NC). This study and all included analyses were approved by Health ABC and the UCSF Committee on Human Research.

## Results

### Statistical appearance model training

Fig 1(a) shows the 82 points used to describe the outline of the body and some key landmarks on the skeleton. Fig 1(b) shows the associated triangulation scheme used to warp the image to a reference frame. Fig 1(c) shows the 52-point subset that excludes the points associated with the lower arms and legs. Fig 1(d) shows the associated triangles to the 52 points. Wherever possible, the triangles in the 52-point annotation are unchanged from the 82-point annotation. This demonstrates how our algorithm can select how the image is warped by manually defining the triangle relationships.

Table 1 shows the relevant demographic and anthropometric markers for the sample participants included in this study. Fig 2 shows the mean image of the 200 calibration participants with progressively more sophisticated registration: (a) translating the images so that the centres of gravity coincide, (b) applying an affine transformation so that the bounding boxes coincide, and (c) using the full piece-wise affine transformation from triangulated mesh. The final registration has corrected for a range of body positions and shapes to bring all the pixels into approximate correspondence, allowing analysis of equivalent structures to be done easily. The images are displayed using the histogram equalised R-images. The models are built from 200 examples and their reflections (400 samples in total).

**Table 1:** Demographic characteristics of selected participants.

Variable	White Men	Black Men	White Women	Black Women	P-value
Calibration set	N = 51	N = 49	N = 50	N = 50	
Age at baseline (yrs)	75.7 (3.0)	73.3 (2.7)	74.3 (3.0)	74.2 (3.1)	<0.01
Height (cm)	172.8 (5.8)	172.1 (5.8)	158.9 (5.2)	158.9 (6.6)	<0.01
Weight (kg)	80.7 (12.8)	77.5 (17.0)	66.0 (12.6)	71.6 (13.1)	<0.01
BMI (kg/m^2^)	27.1 (4.3)	26.1 (5.3)	26.2 (5.0)	28.4 (4.9)	0.07
BMI Category (n)					
Underweight	1	5	4	0	
Normal	16	16	14	10	
Overweight	22	16	26	26	
Obese	12	12	6	14	
6-year status (n)					
Living	26	24	25	25	
Deceased	25	25	25	25	
Sagittal diameter (cm)	22.8 (3.2)	21.9 (3.7)	21.1 (2.8)	22.7 (2.8)	0.03
Abdominal circ. (cm)	101.1 (10.7)	96.4 (14.8)	96.4 (11.2)	97.7 (12.3)	0.18
Validation set	N = 50	N = 50	N = 50	N = 50	
Age at baseline (yrs)	74.7 (3.2)	72.4 (2.6)	74.0 (2.5)	74.1 (2.8)	<0.01
Height (cm)	172.2 (5.4)	172.7 (7.3)	159.8 (5.4)	159.8 (6.2)	<0.01
Weight (kg)	76.6 (9.8)	78.7 (14.7)	65.4 (13.5)	72.6 (11.9)	<0.01
BMI (kg/m^2^)	25.8 (2.9)	26.3 (4.3)	25.7 (5.6)	28.5 (4.9)	0.08
BMI Category (n)					
Underweight	0	0	2	0	
Normal	24	20	22	14	
Overweight	20	22	16	20	
Obese	6	8	10	16	
6-year status (n)					
Living	25	25	25	25	
Deceased	25	25	25	25	
Sagittal diameter (cm)	21.8 (2.6)	22.2 (3.3)	20.6 (3.4)	22.4 (3.3)	0.02
Abdominal circ. (cm)	98.9 (9.2)	97.1 (12.5)	97.2 (14.7)	97.1 (11.9)	0.86

400 total participants in a stratified case-control design were split evenly into training and validation sets. Continuous variables are reported as [mean (standard deviation)]. P-values reported are for overall group differences (one-way ANOVA).

**Fig 3:**
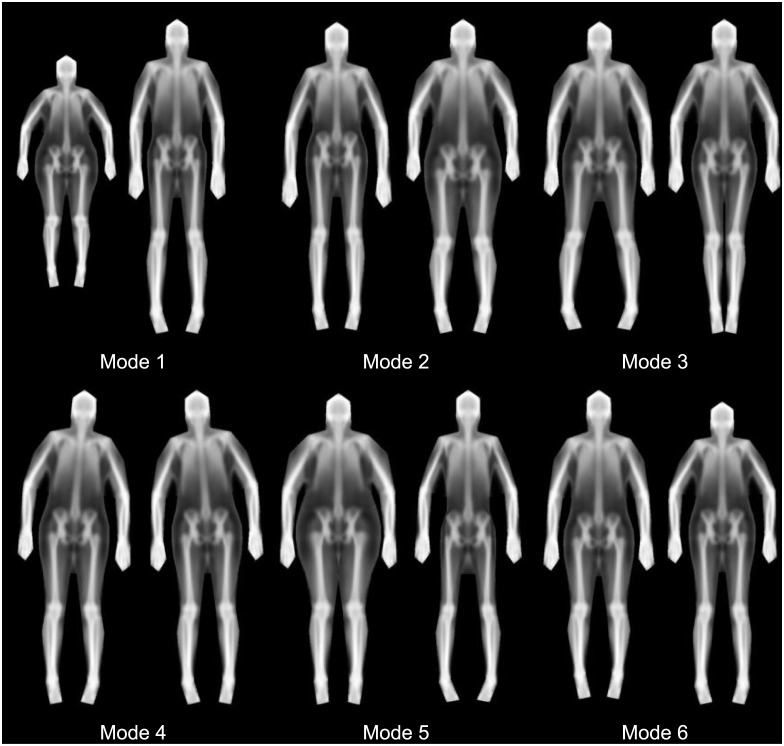
Shape only modes (first 6). For each mode, the -/+ 3 standard deviation (left and right respectively) images are shown. 23 modes were required to explain 95% of shape variance. At a high level, we see that body height is captured in Mode 1, width in Mode 5, and android/gynoid shape variation in Mode 2. Note that several modes capture variation in subject pose.

**Fig 4:**
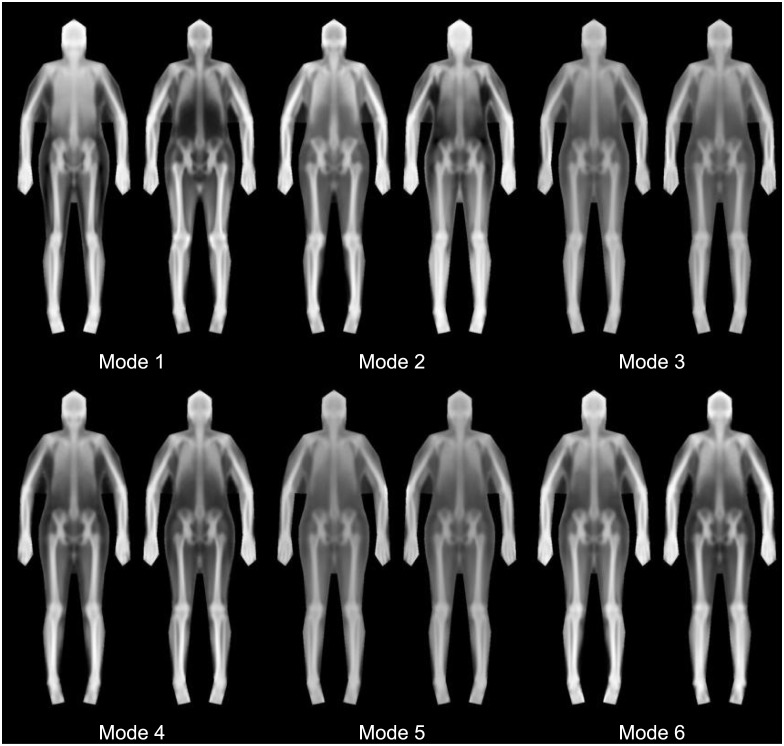
Texture only modes (first 6). For each mode, the -/+ 3 standard deviation (left and right respectively) images are shown. 261 modes explained 99% of the variance. Since this model captures only texture information, all images have the same shape, but differing grayscale intensity indicating different distributions of tissues throughout the regions of the body.

**Fig 5:**
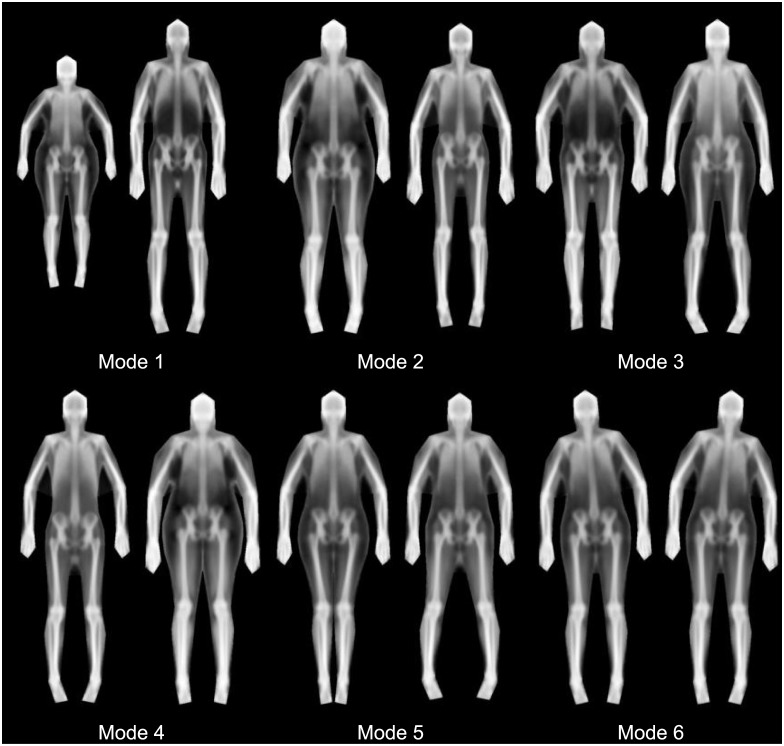
Combined appearance modes (first 6). For each mode, the -/+ 3 standard deviation (left and right respectively) images are shown. 237 modes explained 99% of the variance. This model captures dominant modes of variation in both texture and shape.

We found that 23 shape modes explained 95% of the shape variance defined by our markers. The first 6 shape modes are shown in Fig 3. Furthermore, after registering all images to the average shape, we found that 261 texture modes explained 95% of the variance in X-ray attenuation (represented as greyscale.) Six texture modes are shown in Fig 4. Fig 5 shows the combination of the shape and texture variances to form the full statistical appearance model. The first 237 SAM modes explained 95% of the combined shape and texture appearance. The model is capable of synthesizing both in-plane shape changes and intensity changes, and shows the main correlations between the two.

### Alternative representations of the statistical appearance model

**Fig 6:**
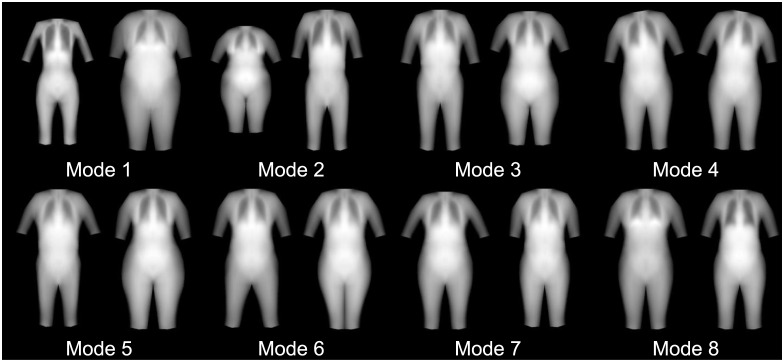
The first 8 appearance modes for a SAM of solid body thickness (lean + fat thickness) and 52-point annotation. **(-/+ 3 SD).** We see significant differences in body shape roughly corresponding to weight, height, and sex in Modes 1, 2, and 3, respectively. Again, pose variation is captured in multiple modes.

**Fig 7:**
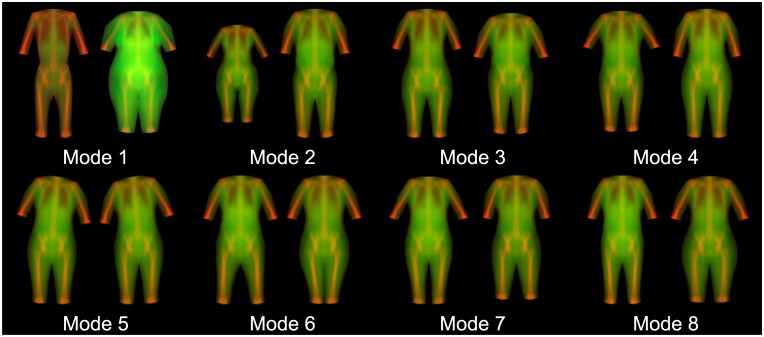
Hybrid model of shape, thickness, and leanness including bone. Total thickness is represented by green intensity and %lean by red intensity. (-/+ 3 SD). Mode 1 captures dramatic body shape and composition variance ranging from a high-lean, low-thickness (thin) phenotype to a low-lean, high-thickness phenotype.

Several examples are given of how different appearance models can be created from different texture information found in the DXA images. Fig 5 shows the first 6 modes of the R-value images where white represents higher density. Fig 6 shows the first 8 modes of an appearance model of shape and body thickness, using a 52-point annotation excluding the forelimbs. Fig 7 shows a combined appearance model of shape, thickness, and leanness, where thickness is encoded as green and leanness is encoded as red in an RGB image. Linear scaling was applied to ensure the data range was in 0 to 255 range. The blue channel was not used.

### Descriptive models

**Fig 8:**
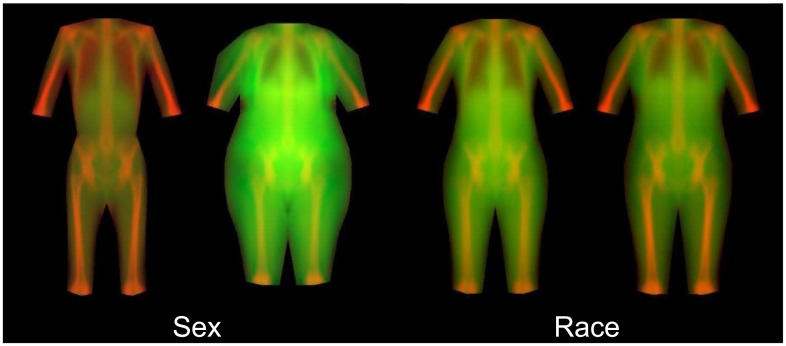
Linear regression models of sex and ethnicity using the combined appearance model of leanness + thickness images (±3 S.D.s). There were 3 appearance modes used in the sex model that achieved an AUROC of 0.99. There were 6 appearance modes used in the Race model that achieved an AUROC of 0.91. These models show that statistical appearance of body shape, thickness, and leanness accurately identifies sex and race differences in the sample population.

**Fig 9:**
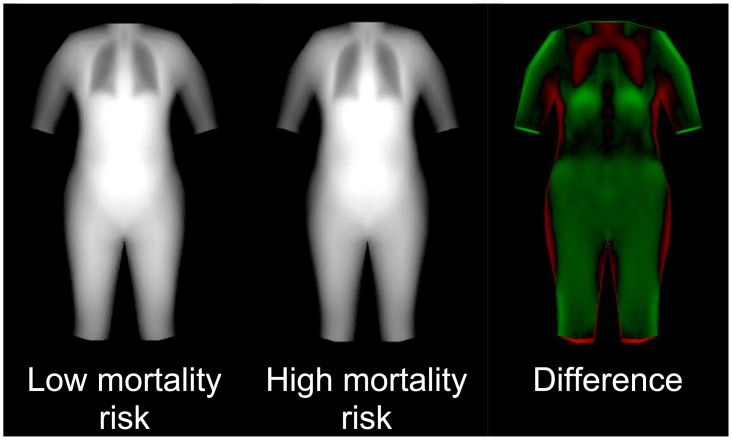
Linear regression model of mortality status using the combined appearance model of thickness images (±3 S.D.s). There were three appearance modes used in the model that achieved training AUC of 0.66 and validation AUC of 0.62. In the difference image, green indicates greater thickness in the low-risk image and red indicates greater thickness in the high-risk image.

Bivariate correlation coefficients between demographic and anthropometric variables and shape modes are found in Table 2. Of these variables, we found that only height predicted sex (AUC = 0.95). Body thickness and leanness, however, was more strongly predictive of sex—the final logistic model includes three shape modes (Table 3) and achieved AUC = 0.99. No combination of the following anthropometric or demographic variables (of sex, BMI, height, weight, sagittal diameter, nor abdominal circumference) predicted race even though this may not be universally true in all datasets. However, body thickness and leanness was a strong predictor of race—the final logistic model includes six shape modes (Table 3) and achieved AUC = 0.91. Visualizations of sex and race models are shown in Fig 8. Using a statistical appearance model of body thickness on the calibration dataset, we found that a logistic model with three SAM parameters predicted mortality with AUC = 0.66. Example images of low- and high-risk body appearances are shown in Fig 9. Note that the primary differences between the low and high risk were the apparent lung volume and waist shape. The mortality model had an AUC = 0.62 when applied to the validation dataset. Regression equations for sex, race, and mortality are provided in Table 3.

**Table 2:** Correlation coefficients for each principal component of the shape model versus demographic and anthropometric variables.

	PC1	PC2	PC3	PC4	PC5	PC6	PC9	PC10
**Age**								
**Sex**	-0.19	0.69	-0.39		0.35			
**Race**		0.23	0.37			0.22	-0.21	-0.26
**BMI**	0.93	0.27						
**Height**	0.22	-0.89	0.29					
**Weight**	0.95	-0.24					0.18	
**Sagittal diameter**	0.89	0.16		-0.16				-0.16
**Abdominal circ.**	0.86			-0.24				

These components correspond to the images in Fig 3. Only correlations with *P* ≤ 0.05 are shown. Bold denotes *P* ≤ 0.01 and shading denotes *P* ≤ 0.01.

**Table 3:** Logistic regression equations for sex, race, and mortality.

Model Type	Outcome	Equation	*α*	Train AUC
Thickness + leanness*	Sex	P(male) = (1+e^-α^)^-1^	-0.178–0.0026pc1 + 0.0041pc2 + 0.0027pc3	0.99
	Race	P(white) = (1+e^-α^)^-1^	0.130–0.0019pc3–0.0008pc4–0.0019pc6–0.0009pc7–0.0013pc8 + 0.0042pc16	0.91
Thickness**	Mortality	P(deceased) = (1+e^-α^)^-1^	0.0336–0.0015pc10 + 0.0014pc11–0.0026pc23	0.66

Parameters for each model were selected using stepwise regression with a required P-value of 0.05 to stay in the model.

*Visual representations of the first 8 thickness + leanness appearance modes are shown in Fig 8.

** Fig 7 shows the first 8 thickness appearance modes.

## Discussion

We have developed methods to describe and analyze the rich regional body shape and composition information captured in whole-body DXA images. We applied statistical appearance modeling techniques to body thickness and leanness images derived from raw DXA attenuation data. The resulting SAM principal components describing holistic body shape were shown to be highly predictive of race and sex, indicating that this technique is capable of distinguishing the unique shape characteristics of each group. Importantly, appearance modes of body thickness were predictive of mortality status. Inspection of the body shape differences captured by the appearance model (Fig 9) reveals interesting features such as apparent lung volume that differ by mortality status. These results suggest that this technique could be used to elucidate body shape and composition phenotypes that may be strongly associated with health status, provide new metrics for risk assessment in individuals, and reveal body features worthy of further research.

Previous work in this area was performed by Wilson in his PhD dissertation [34]. Wilson created whole body principal component models that used only rigid affine-aligned thickness images. This work did not include piecewise registration, or other image types. The preliminary models had a blurry appearance, similar to Fig 2(b), due to the lack of precise registration. Nonetheless, Wilson was still able to show strong correlations to patient demographic variables. Later, Wilson showed that body shape was related to mortality using trunk to leg volume ratios from DXA images [35]. In his fully adjusted models for mortality, he demonstrated strong AUC values of 0.83. Besides the representation of body shape, the Wilson study design differed from our design in population (Wilson: NHANES 1999–2004, ages 20 to 85 years; Health ABC: 75 years at baseline) and adjustments (Wilson: Age, gender, race, BMI, waist circumference, activity level, poverty index; Health ABC: none). Further future evaluations are planned in the NHANES population Wilson used to directly compare the SAM methods directly to simple measures like trunk to leg volume ratio.

Shape and appearance modeling has been applied to proximal femur DXA scans with success [22, 36, 37]. Goodyear et al. [37] showed that the combination of shape and appearance models with bone density produced the best AUC = 0.65 compared to any single measure for predicting hip fracture risk. To our knowledge, this is the first application of SAM techniques to whole body DXA images. The models for sex, race, and mortality risk derived herein demonstrate the potential of this approach to provide novel and significant image features from standard DXA data.

This study had notable strengths. First, there was a similar number of men and women, and black and white participants. This is important because the models derived are equally weighted by sex and ethnicity. Second, because of our case and control design, we were able to increase the signal present in the model for mortality over what would be expected in a prospective study of the same number of participants. However, this study had some limitations. First, the DXA data was acquired on one make of DXA system (Hologic). Our statistical appearance models would not be applicable to other makes without further validation. Additionally, the study population was limited to a narrow age range. A more complete analysis of body shape and appearance in a broader, representative sample of adults is warranted to ensure generalizability. Another issue was the limited data available for training the constrained local model for automatic annotation of the DXA images. All images required some degree of manual annotation point adjustment where the automated placement algorithm did not accurately detect body landmarks. Given sufficient high-quality training data, though, the automated CLM technique has been shown to achieve very good accuracy [28]. We expect that a large training dataset of DXA images across a wide range of body shapes and compositions would yield a precise and accurate active appearance model for fully-automated annotation.

Detailed models of the body shape and tissue distribution offer significantly more information than standard DXA analyses. This study demonstrates a method for describing holistic body shape, thickness, and leanness that reveals unique features by sex, race, and also predicts mortality risk. Further study is warranted to investigate body shape associations to other outcome variables of interest, across different populations. As this technique utilizes standard whole body DXA image data, it is readily applicable to several existing study databases of DXA scans. In addition, supervised methods of feature selection beyond principal component analysis may yield more sensitive and specific predictors for clinical outcomes.
